# Positive Psychological Attributes and Entrepreneurial Intention and Action: The Moderating Role of Perceived Family Support

**DOI:** 10.3389/fpsyg.2020.546745

**Published:** 2020-12-08

**Authors:** Martin Mabunda Baluku, Julius Fred Kikooma, Kathleen Otto, Cornelius J. König, Nida ul Habib Bajwa

**Affiliations:** ^1^ Department of Educational, Social, and Organizational Psychology, School of Psychology, Makerere University, Kampala, Uganda; ^2^ Department of Work and Organizational Psychology, Faculty of Psychology, Philipps-University Marburg, Marburg, Germany; ^3^ Department of Work and Organizational Psychology, Faculty of Human and Business Sciences, Saarland University, Saarbrücken, Germany

**Keywords:** proactive personality, psychological capital, entrepreneurial intention, implementation intention, perceived family support, entrepreneurial action

## Abstract

Recent research illustrates substantial gaps between entrepreneurial intentions and behavior. This is a challenge for entrepreneurship promotion interventions that have primarily focused on stimulating entrepreneurial intentions. However, extant literature suggests that implementation intentions enhance the likelihood of acting congruently to the behavioral intention. Furthermore, theory also suggests the condition effects of situations and the perceived control over them. We therefore hypothesized that implementation intentions mediate the relationship between entrepreneurial intention and action, while perceived family support moderates the movement from implementation intention to entrepreneurial action. Using two-wave survey data from a sample of students at an African university, we measured two psychological attributes (proactive personality and psychological capital) as important precursors of entrepreneurship and entrepreneurial intentions present before undertaking an innovations and entrepreneurship course. Implementation intentions regarding entrepreneurship, entrepreneurial actions, and perceived parental support for entrepreneurial activities were also measured 2 weeks after completion of the course. Our results demonstrate support for the proposed moderated double mediation model in which the effects of the two psychological attributes on entrepreneurial actions are explained *via* entrepreneurial intentions and implementation intentions. We further find moderation effects of perceived family support indicating that implementation intentions more likely predicted entrepreneurial actions in cases of higher family support.

## Introduction

There has been increased attention to stimulating entrepreneurial intentions among youth, particularly among university students, through entrepreneurship education. Entrepreneurial intentions refer to the state of mind that directs one’s attention, experience, and action toward a specific entrepreneurial behavior (Bird, 1988; Obschonka et al., 2010). Universities and government policies in many countries are gearing toward entrepreneurially-oriented graduates who view self-employment as a feasible career path (Nabi and Holden, 2008). Consequently, research on entrepreneurial intentions is advancing rapidly (Liñán and Fayolle, 2015) and entrepreneurship education is one of the fastest growing academic fields (Nabi et al., 2017). Especially in developing countries, there are great expectations from policymakers that relate to start-ups by university students. Additionally, recent graduates are regarded as important for economic growth and development through utilization of their high human capital. Similarly, as unemployment rates among youth are very high in these countries and the level of industrialization is typically low, entrepreneurship is seen as a viable means of enabling young people to avoid unemployment after graduation (Gorgievski et al., 2018) and generating jobs in local economies (Ayyagari et al., 2011).

Entrepreneurship is increasingly becoming an important career path for many individuals around the world, especially when placed in the context of income inequalities and extremely high youth unemployment rates, especially in the Sub-Saharan Africa (Ackah-Baidoo, 2016; Sulemana et al., 2019), Although a majority of the start-ups are small in size, entrepreneurship is at least a means to obtaining a job and earning livelihoods (Gindling and Newhouse, 2014; Falco and Haywood, 2016). In countries like Uganda, a large percentage of graduates cannot be absorbed into available job openings (Baluku et al., 2020). Consequently, governments and their development partners have placed special emphasis on entrepreneurship education as a strategy for boosting business start-ups, job creation, and alleviating poverty.

Complementing the Theory of Planned Behavior (TPB) with ideas from positive psychology, we focus on the contribution of positive psychological attributes (specifically proactive personality and psychological capital) to the formation and implementation of entrepreneurial intentions. Proactive personality describes an individual that is not constrained by situational forces but rather takes action to make changes in their environment (Bateman and Crant, 1993; Crant, 1996); while psychological capital is a kind of capital that defines an individual’s psychological strength and comprises four resources including confidence, hope, resilience, and optimism (Luthans et al., 2004). The TPB model presents such attributes among background factors that have a distal and indirect influence on intentions through their effects on behavioral beliefs, normative beliefs, and control beliefs (Ajzen and Albarracin, 2007).

Entrepreneurship education often targets entrepreneurial intentions with the view that this results in the establishment of firms (Liñán, 2004). Understanding the role of such important distal factors can be useful in improving entrepreneurship education programs. Moreover, researchers have recently illustrated that there are substantial gaps in the association between entrepreneurial intentions and behavior (van Gelderen et al., 2018). In fact, this research suggests that even strong intentions do not necessarily predict entrepreneurial behavior. Hence, there have been questions about the relevance of research that only focuses on entrepreneurial intentions (van Gelderen et al., 2018). At the same time, in developing countries such as Uganda, high levels of entrepreneurial intentions can be found (GEM, 2014; Singer et al., 2015; Baluku et al., 2019b). Yet, only a limited number of entrepreneurs have actually succeeded in creating a viable business in the past (Vivarelli, 2013). Consequently, there is a need to investigate the mechanisms that link entrepreneurial intentions and behavior and other mechanisms that moderate this relationship (Nabi et al., 2017), in order for entrepreneurship education to be designed in an impactful way.

One mechanism that has been suggested to mediate the relationship between intentions and entrepreneurial behavior is the forming of implementation intention (van Gelderen et al., 2018; Tornikoski and Maalaoui, 2019). Although some entrepreneurship studies have recently focused on the role of implementation intentions using datasets from developed countries (van Gelderen et al., 2018; Esfandiar et al., 2019), there is a paucity of studies focusing on the role of implementation intentions in the entrepreneurial processes of developing countries. Implementation intentions refer to the plan of how, where, and when to perform a behavior (Gollwitzer, 1993, 1999). Furthermore, there is an increasing realization by researchers across the world that national and cultural differences can play an important role in the understanding of psychological concepts. This is highlighted by a heavy reliance on research samples from developed countries (Henrich et al., 2010; Van de Vliert and Van Lange, 2019). For example, African societies can be characterized by a greater focus on starting businesses out of necessity instead of seeking out innovation opportunities and on taking care of relatives and the community (McElwee, 2009), thereby having an impact on the actual entrepreneurial behavior. Similarly, given the relatively high odds of failing with a business idea in Africa (Balunywa et al., 2013; Nangoli et al., 2013), proactivity in pursing entrepreneurial endeavors might be impacted (Kiggundu, 2002; Ladzani and van Vuuren, 2002).

Therefore, using a sample from Ugandan university students, we focus on positive psychological attributes including proactive personality and psychological capital as distal antecedents that reveal both direct and indirect effects on entrepreneurial intentions and behavior. At the same time, the context plays an important conditioning role in the implementation of entrepreneurial intentions, although extant research is relatively silent on contextual influences on the translation of intentions into start-up behavior (Weiss et al., 2019). If understanding intentions is to be useful, it is a necessary step to further explore how and when entrepreneurial intentions translate into entrepreneurial start-ups or actions. In the present study, we focus on family support for entrepreneurial activities as a contextual and potentially boosting factor of entrepreneurial implementation intentions that is likely to be particularly relevant in a rather collectivistic society (Sawitri et al., 2014) such as Uganda.

### Theory and Hypothesis Development

Establishing a new venture is a particularly complex process involving a large set of activities and behaviors. Prospective entrepreneurs have to identify a problem, find a team to work on a business opportunity, conduct thorough market analysis, create a product or service, navigate through the legal process of establishing the firm, are confronted by highly dynamic business environments that demand quick decision making, bear the risk of likely losses, and are challenged by harnessing financial and non-financial resources required for start-ups (Grandi and Grimaldi, 2005; Baron et al., 2016). At the same time, the risks of failure are quite high with the overwhelming majority of entrepreneurial ideas failing to have an impact on the market for a prolonged period of time (Shane, 2010). Nonetheless, there are many people who are willingly investing their time and efforts to pursue entrepreneurial thoughts and ideas across the world.

Despite the challenges in starting new businesses, economies around the globe need more entrepreneurs. The complexities of the labor market that include high unemployment, job insecurity, and shifts in economic systems have resulted in greater emphasis on entrepreneurship or self-employment as a feasible work alternative (Baluku, 2017). These have a net effect on what kind of jobs are available, how fast people can transit from school-to-work, and where people work. Moreover, the movement from industry-led economies to information and service driven ones have provided numerous entrepreneurial opportunities enabling individuals to transform their intelligence, innovative abilities, and imaginations into job creation and wealth generating activities (Baluku, 2017). At the macro level, entrepreneurship is important for economic resilience and the development of regions and nations (Williams et al., 2013; Huggins and Thompson, 2014).

Although the need for more entrepreneurs and start-ups is a global necessity, developing countries might face greater challenges as high unemployment rates drive many young people to create their own business out of necessity rather than primarily focusing on opportunities (Magelah and Ntambirweki-Karugonjo, 2014; Baluku, 2017). Furthermore, prospective entrepreneurs are confronted with challenges related to limited access to financial capital and markets; shortages of basic resources needed for doing business, such as insufficient or irregular electricity; and a general lack of a supportive entrepreneurial ecosystem (Asongu and Odhiambo, 2019). For example, nascent entrepreneurs in Africa tend to have limited business support and mentoring in terms of counseling, marketing services, and accessing useful networks (Amha and Ageba, 2006). Consequently, the process of starting up a company is marred by numerous potential stressors (Baron et al., 2016).

So far, research on this topic focuses on better understanding factors facilitating the creation of companies from numerous fields such as economics, management, or psychology (Landström and Lohrke, 2010; Frese and Gielnik, 2014). For example, studies in the field of economics try to better understand the economic impact that entrepreneurial activities can have on the local, regional, national, or global levels of unemployment (Landström and Lohrke, 2010). Whereas, studies in management might try to identify differences in marketing strategies and their likely success in different international markets (Hills and Laforge, 1992). Although these perspectives might already elicit areas that individuals could work on to improve their chances of creating a successful business, in many cases research wants to understand how entrepreneurship develops and therefore needs to focus on people that have not yet started their own company but intend to do so in the future. To this purpose, including psychological perspectives to entrepreneurship is necessary (Frese and Gielnik, 2014).

The study of entrepreneurial intentions has become a very important stream within entrepreneurship research. It seeks to understand the underlying factors for people to start their own businesses, since intentions have been found to predict subsequent entrepreneurial behavior (Krueger, 2017; Donaldson, 2019). This stream of research has heavily relied on the TPB (Ajzen, 1985, 1991), since entrepreneurial behavior is largely volitional and planned, and hence tends to follow intentions that develop in a period of time. According to the TPB, entrepreneurial intentions are a function of a belief system comprising of behavioral beliefs, normative beliefs, and control beliefs (Ajzen, 1991; Ajzen and Albarracin, 2007). This belief system can be translated into three antecedents of intentions including attitude toward the behavior, subjective norms, and the perceived behavioral control, respectively (Ajzen, 1991; Tornikoski and Maalaoui, 2019). Hence, intentions are a mediator of the link between the antecedent factors and the respective behavior (Kautonen et al., 2015). The theory acknowledges that the beliefs that translate into the three proximal antecedents of intentions also develop from a wide range of dispositional, demographic, and informational or contextual factors (Ajzen, 1991; Ajzen and Albarracin, 2007). From the dispositional individual differences, we focus on proactive personality and psychological capital. In addition, we suggest that perceived family support is one of the conditions that are useful when it comes to the implementation of entrepreneurial intentions. Aspects of perceived family support may enhance both perceived behavioral control and subjective norms.

Past research has relied on measuring the intent to start a business during and after entrepreneurial education programs that seek to boost entrepreneurial efforts of participants (Bae et al., 2014). This causal flow from intentions to behavior (e.g., Barbosa et al., 2008; Kautonen et al., 2013; Van Gelderen et al., 2015; Baluku et al., 2019c; Weiss et al., 2019) has recently been put into question by research that illustrates a gap between intentions and actions. This is because not everyone with entrepreneurial intentions starts a business venture (Shirokova et al., 2016; van Gelderen et al., 2018; Tornikoski and Maalaoui, 2019; Weiss et al., 2019). At the same time, research has also found that environmental context factors play a crucial role in the intention-action link of entrepreneurship. The same research calls for a better and more global understanding of the intention-action link of entrepreneurship (Shirokova et al., 2016; Weiss et al., 2019).

Although a small number of studies have questioned the translation of entrepreneurial intentions into entrepreneurial behavior, these studies have only been conducted in countries with comparatively lower entrepreneurial intentions among its overall population (Adam and Fayolle, 2015; van Gelderen et al., 2018). In East African countries, such as Uganda, there is a pronounced difference between the overall levels of entrepreneurial intentions and early stage entrepreneurial activities (GEM, 2014; Singer et al., 2015). This makes it an ideal place to study the discrepancy between entrepreneurial intentions and actions. The reasons for a limited understanding of the entrepreneurial intention and action link might be very basic, as research in many domains of psychology has been shown to mostly utilize student’s samples from Western, Industrialized, Rich, and Democratic countries (WEIRD) and implicitly assuming that findings from WEIRD samples generalize to the world population (Henrich et al., 2010). For applied fields of research, such as entrepreneurship psychology, it is especially important to make impactful research (Devine and Kiggundu, 2016). Given the scarcity of research on the entrepreneurship intention and behavior link, this study seeks to build upon prior research that only utilized WEIRD samples and to apply previous knowledge in the East African context.

### Proactive Personality

Personality is one of the major domains in entrepreneurship psychology research given its contribution to understanding and predicting human behavior across different settings. Although extant research seems to draw from the so-called WEIRD populations, personality could also be essential in explaining entrepreneurial intention and behavior in less studied setting such as those in low-income countries. The personality approach has been heavily criticized because observed traits are too broad to predict specific behaviors like entrepreneurial behavior, Rauch and Frese (2007a) argued that there are avenues for enhancing its contribution to the study of entrepreneurship. Broad personality traits specifically comprise of the Big Five, which tend to explain broad or general behaviors across different settings (Loveland et al., 2005). To enhance the contribution of personality to understanding entrepreneurship, there have been calls to shift the focus from broad personality traits to narrow ones in predicting entrepreneurial outcome variables since they are likely to be more proximal to behavior (Obschonka and Stuetzer, 2017; Arco-Tirado et al., 2019; Obschonka et al., 2019). Narrow traits are less broad in their scope and tend to account for greater variance in work related outcomes (Loveland et al., 2005), and hence could be more important to the development of entrepreneurial mindsets and closer to entrepreneurial behaviors (Rauch and Frese, 2007a,b; Schmitt-Rodermund et al., 2017). At both the broad and narrow perceptions of traits, each personality characteristic represents a set of interests, preferred activities, beliefs, abilities, values, and characteristics (Nauta, 2010). Brandstätter (2011) argues that personality may reveal a stronger influence in entrepreneurship than in any other profession. In our study, we focus on the proactive personality since entrepreneurs are described as adventurous, acquisitive, ambitious, confident, and sociable (Spokane and Cruza-Guet, 2005). These qualities are characteristics of proactivity.

**Figure 1:**
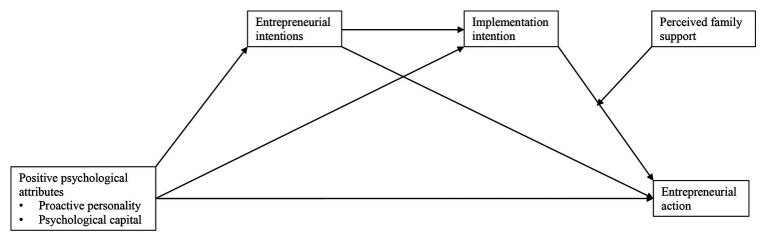
Conceptual model.

A proactive personality refers to the predisposition of people to exhibit behaviors which involves taking actions to influence their environments (Bateman and Crant, 1993; Crant, 1996). Proactive behaviors include actions such as creativity, initiative, networking, taking control, knowledge of power structures, adaptability, perseverance, and resilience (Crant, 1996; Seibert et al., 2001; Fuller and Marler, 2009). Proactive people can identify opportunities from their environments and take the required steps to seize those opportunities. These behaviors are useful at different stages of the entrepreneurial process. Empirical studies linked a proactive personality to entrepreneurial motivation and intentions (Crant, 1996; Chan et al., 2015; Mustafa et al., 2016; Neneh, 2019b). It is also associated with the likelihood of starting up new ventures (Becherer and Maurer, 1999). People with a strong proactive personality not only develop an intention to seize entrepreneurial opportunities in their environment, but also act on those opportunities (Brandstätter, 2011; Neneh, 2019a). Although environmental factors in developing countries, such as Uganda, can often hinder proactive behaviors (e.g., having higher failure rates due to economic conditions and a limited scope for certain ideas), it is plausible to assume that proactivity as a personality trait should nonetheless be linked to entrepreneurial intentions and actions. This is because they are found in studies in developed countries (Obschonka et al., 2018; Neneh, 2019a). Consistent with this literature, our first hypothesis focuses on replicating these relationships in the Ugandan context (see also Figure 1 for the full model):

Hypothesis 1: A proactive personality is positively associated with (a) entrepreneurial intentions and (b) entrepreneurial actions.

A second precursor of entrepreneurship is entrepreneurial psychological capital (Pease and Cunningham, 2016; Pease et al., 2018). Rooted in positive organizational psychology (Luthans, 2002; Luthans et al., 2004; Luthans and Youssef, 2007; Luthans and Youssef-Morgan, 2017), psychological capital is conceptualized as a positive developmental state encompassing confidence (self-efficacy), hope, optimism, and resilience. These psychological resources are related such that an increase in one is likely to result in the increase in the other (Luthans and Youssef-Morgan, 2017). Together, they enable people to have a sense of control when pursuing goals (Nolzen, 2018).

Self-efficacy is one of several individual resources that have already been linked to entrepreneurship. These resources are important in the formation of intentions and actions that lead to start-ups. Some other resources include opportunity recognition, seeking start-up resources, and lowered perceptions of risk and the likelihood of failure (Goel and Karri, 2006; Dimov, 2010; Culbertson et al., 2011; Piperopoulos and Dimov, 2015; Contreras et al., 2017). Optimism relates to outcome expectations which are essential in the formation of behavioral attitudes and intentions (Ajzen, 1991). These psychological resources are also critical in recognizing and evaluating business opportunities, and hence influencing start-up decisions (Trevelyan, 2008; Rigotti et al., 2011; Storey, 2011). The psychological resources of hope and resilience are relevant to achieving the level of persistence required for entrepreneurial activities. Hope enables the development of behavioral goals and persistence in actions for achieving those goals (Snyder, 2002; Luthans et al., 2007c). Additionally, it enables coping with challenges and uncertainty (Luthans et al., 2007a,b). Consequently, entrepreneurs are able to cope with the tough challenges involved in their job (Baron et al., 2016; Bockorny and Youssef-Morgan, 2019).

Psychological capital, and its dimensions seem to have a strong influence on the formation of entrepreneurial intentions (e.g., Contreras et al., 2017; Baluku et al., 2019c). For example, having optimism that available opportunities will lead to desired outcomes is likely to stimulate action. Consequently, psychological capital is useful in entrepreneurial perceptions (Worthington and Kasouf, 2018) that are important in opportunity recognition and start-up decisions. Psychological capital has also been found to play an important role in realizing the effects of motivational variables such as business support, mentoring, empowerment, and courage on entrepreneurial outcomes (Newman et al., 2014; Baluku et al., 2019c; Bockorny and Youssef-Morgan, 2019; Digan et al., 2019). In addition, psychological capital is associated with the entrepreneurship process and outcome variables such as business leadership, persistence, and performance (Jensen and Luthans, 2006; Hmieleski and Carr, 2008; Baluku et al., 2018b). The control aspects of psychological capital (Nolzen, 2018) are relevant at all stages of the entrepreneurial process. Finally, given the adverse conditions under which entrepreneurial endeavors are started in developing countries, in addition to bureaucratic ease in those countries (especially as entrepreneurial efforts are often started informally), it is likely that we can replicate the previously mentioned relationships and findings from studies in developed countries in our second hypothesis:

Hypothesis 2: Psychological capital is positively associated with (a) entrepreneurial intentions and (b) entrepreneurial actions.

Up until this point, the hypotheses aim to replicate existing knowledge in entrepreneurship psychology research from developed countries (e.g., Shirokova et al., 2016; Contreras et al., 2017). In East African countries, such as Uganda, where entrepreneurial intentions are often found to be extraordinarily high, there is a lack of understanding on why entrepreneurial intentions do not translate into actual entrepreneurial behavior (Gielnik et al., 2014). This is especially important not only for these countries themselves, but also for the research community as the level of industrialization is typically low in such economies, and finding jobs in the industry is rather uncommon (Falco and Haywood, 2016; Baluku et al., 2020). This creates a substantially higher demand for entrepreneurs to succeed creating jobs for themselves as well as for others. This demand partly stems from strategic misalignments of the needs of the industry and current education programs offered by higher education institutions in East Africa. Institutions create many graduates with higher education degrees for which there is little demand in the job market (Kristensen and Birch-Thomsen, 2013). Given the obvious barriers of entering the job market, it is plausible that students and recent graduates exhibit high entrepreneurial intentions (Baluku et al., 2019b). This makes activities for increasing entrepreneurial intentions (a cornerstone in entrepreneurial education efforts in developed countries) rather subordinate to activities that would help utilize the strong entrepreneurial intention foundation already present in East African students, while moving them toward entrepreneurial behavior. Therefore, we present two hypotheses in the following sections that could help explore this relationship.

### Implementation of Entrepreneurial Intentions as a Mediating Mechanism

Recent research by van Gelderen et al. (2018) has found implementation intentions to be critical in better understanding the link between entrepreneurial intentions and entrepreneurial behavior. The concept of implementation intention represents the how, when, and where of actions leading to the achievement of the desired goal (Gollwitzer, 1999). In an entrepreneurial sense, implementation intentions involve making concrete plans on how to start a new business venture (Tornikoski and Maalaoui, 2019). Having implementation intentions, in terms of automatic action initiation, facilitates performing the desired behavior even in the presence of cognitive barriers that lessen the level of control to achieving goal intentions (Gollwitzer, 1993; Brandstätter et al., 2001). Another view of implementation intention is that it generates a commitment to perform the target behavior (Ajzen et al., 2009). Both views tend to suggest that implementation intentions are a mediating link between behavioral intentions and performance of the behavior. Empirical studies in the field of entrepreneurship support this assumption (Adam and Fayolle, 2015; van Gelderen et al., 2018). We propose that a proactive personality and psychological capital are associated with entrepreneurial intentions and action. Considering this literature, we further hypothesize mediated mediations such that:

Hypothesis 3a: The effects of a proactive personality on entrepreneurial actions are mediated by entrepreneurial intentions and implementation intentions.
Hypothesis 3b: The effects of psychological capital on entrepreneurial actions are mediated by entrepreneurial intentions and implementation intentions.

### The Role of Family Support

Although contextual variables seem important for the implementation of entrepreneurial intentions, this has rarely been analyzed in the literature so far (Weiss et al., 2019). Contextual factors are important because they might have the ability to boost or diminish intentions. For example, they can provide resources or eliminate barriers to running a business (Tornikoski and Maalaoui, 2019). Apart from broader contextual factors such as culture or regional specificities, effects of family support on entrepreneurship have not been focused on in the past. Yet, there is a higher reliance on communities and families for businesses to thrive, especially in developing countries where most of the business activity is conducted informally (Williams et al., 2017), with emotional and instrumental family support being crucial elements (e.g., Edelman et al., 2016). Emotional family support could be related to indicating approval (a crucial aspect for members of collectivistic cultures) and could reinforce start-up efforts of young entrepreneurs. Support could also arise in terms of giving advice (Shen et al., 2017). For young graduates, parents are known to have a strong influence on the career choices of their children (Sawitri et al., 2014). This creates a relevant influence of family support on the transition from entrepreneurial intentions to entrepreneurial behavior.

In terms of instrumental family support, family is also a major source of start-up capital for nascent and young entrepreneurs. Although, capital provided by the family has been found to not correlate positively with the scope of start-up activities (Edelman et al., 2016). Nonetheless, extant research demonstrates that most small-scale start-ups are financed by family and friends (Aldrich and Martinez, 2007). This is certainly true for less developed and collectivistic societies such as Uganda (Baluku, 2017; Baluku et al., 2019a). Hence, family support is a reaffirmation that the family will support entrepreneurs in obtaining resources, especially when family or personal savings are a major source of start-up capital. Parents can boost the likelihood of a child becoming an entrepreneur through approval of entrepreneurship as a viable career option, encouragement, and approval of their children’s business ideas. Perceived family support provides a motivational force that is particularly important for the implementation of entrepreneurial intentions (Elfving et al., 2017). This plays a role that is similar to that of entrepreneurial mentoring. It has been suggested that parents tend to have the same characteristics of entrepreneurship mentors (Eesley and Wang, 2017). Consequently, perceived or actual parental support can motivate young people to commit and exert effort in implementing their entrepreneurial intentions. We therefore propose that family support is an important conditioning factor when it comes to the implementation of entrepreneurial intentions. This then leads to the following hypotheses:

Hypothesis 4a: The indirect effects of a proactive personality on entrepreneurial actions through entrepreneurial intentions, and implementation intentions are moderated by perceived family support.
Hypothesis 4b: The indirect effects of psychological capital on entrepreneurial actions through entrepreneurial intentions, and implementation intentions are moderated by perceived family support.

## Materials and Methods

### Procedure and Participants

Given the extraordinarily high unemployment rates in Uganda among young graduates (UN Department of Economics and Social Affairs, 2019), only a small number of young graduates are able to secure a job in the formal sector. With almost 74% of the total workforce in Uganda working in rural areas and primarily in the agriculture sector (World Bank, 2012), the job market for young graduates from a wide variety of fields is limited to low productivity opportunities in the informal or formal sectors. This makes starting an own business a relevant career path for most graduates (World Bank, 2012; Falco and Haywood, 2016). We therefore selected students of a third (final) year Bachelor of Industrial and Organizational Psychology program at Makerere University in Uganda. We embedded our research within the evaluation of a course named “innovations and entrepreneurship” to serve as a sample. The course focuses on understanding the application of psychological knowledge to entrepreneurship, the process of establishing and managing business ventures, the skills needed for establishing and managing business ventures such as business plan development, and developing creativity and innovation. Occasionally, former students of the program who have established successful business ventures are invited to give motivational talks to the students.

The evaluation of the course included several outcome variables of entrepreneurial education such as entrepreneurial attitudes and intention, entrepreneurial alertness, opportunity recognition, implementation intention, and entrepreneurial action, among others. We also measured several predictor variables such as proactive personality, psychological capital, social capital, and relational capital. The present paper only focuses on the associations among some of these variables at pre-study (T1) and post-study (T2) evaluations.

Out of the 222 students enrolled in the course, 196 (64.8% female and 35.2% male) voluntarily completed the pre-study evaluation at the beginning of the course (T1). The average age of the respondents at T1 was 23.36 years (*SD* = 3.20). The post-study (T2) evaluation was conducted 4 months after the pre-study evaluation (2 weeks after the end of the course). At T2, 157 students (65% female and 35% male) completed the evaluation questionnaire, and we were able to match 149 responses (representing 76.02% of the participants at T1). The average age at T2 was 23.52 years (*SD* = 3.25). The descriptive statistics of study and control variables of all respondents at T1 and T2 are not statistically different; hence, there was no systematic drop out.

### Measures

#### Independent Variables

The study focuses on the effects of two positive psychological attributes: proactive personality and psychological capital (measured at T1). We measured a proactive personality with the shortened five-item version (Janssen et al., 2017) of the original scale (Bateman and Crant, 1993). A sample item is “I love being a champion for my ideas, even against others opposition” (*α* = 0.86). For psychological capital, we used the short version (PCQ12) of the questionnaire of Luthans et al. (2007a). This questionnaire comprises 12 items (sample: “I can think of many ways to reach my current work/career goals”) and had a Cronbach’s *α* of 0.90. For both independent variables, the short versions of the measures were adopted due to the limited time for assessment (Janssen et al., 2017), given that the evaluation included several other constructs that are not part of the present paper. Responses to both scales were indicated on a six-point Likert scale ranging from “1 = strongly disagree” to “6 = strongly agree.”

#### Mediator Variables

The intention constructs were the mediators in this study. Entrepreneurial intention was measured at T1 using the Liñán and Chen (2009) six-item questionnaire. A sample item is “I have the firm intention to start a firm someday” (*α* = 0.90). At T2, we measured implementation intentions regarding entrepreneurial activities using an established three-item questionnaire (van Gelderen et al., 2018). A sample item is “I have already planned precisely what I will do as my first step to starting a business” (*α* = 0.92). Items in both questionnaires were assessed on a six-point Likert scale ranging from “1 = strongly disagree” to “6 = strongly agree.”

#### Moderator Variable

We measured family support at T2 with six items. First, participants were asked to indicate the extent to which their parents or family are supportive of: (1) them becoming self-employed and (2) their respective business idea on a scale of “1 = not supportive at all” to “6 = very supportive.” In addition, participants were asked to rate the following four items on a six-point Likert scale ranging from “1 = strongly disagree” to “6 = strongly agree”: (3) “My parents/guardians encourage me to become self-employed,” (4) “My parents are okay with me making a career in entrepreneurship,” (5) “Other family members support my idea of becoming self-employed,” and (6) “My parents or someone in the family are willing to provide me with start-up capital.” The ad-hoc developed scale demonstrated acceptable internal consistency (*α* = 0.84).

#### Dependent Variable

Entrepreneurial action was measured at T2 with eight items adapted from Valliere (2015). Participants were asked to what extent they have engaged in the following actions in the past few weeks: (1) “I have developed a business plan with an intention of implementing it,” (2) “I have obtained valuable inputs for the business (e.g., land, business premises/space, raw materials, etc.),” (3) “I have taken steps to open a bank account for the business,” (4) “I have increased amount of time spent on focusing on my specific business idea,” (5) “I have made analysis of how my intended product/service/trade will fare/compete in the market (i.e., made a market feasibility analysis),” (6) “I have registered the business or in the process of registering the business with the relevant authorities,” (7) “I have taken effort to harness/solicit for the required resources to start the business (e.g., start-up capital),” and (8) “I have invested my own resources in the process of starting the business.” The items illustrated a high level of internal consistency (*α* = 0.91).

## Results

Descriptive statistics and correlations among study variables are reported in Table 1. To rule out multicollinearity effects, we computed variance inflation factors that ranged from 1.40 to 1.86. This suggests that there are no collinearity concerns in our data (Hair et al., 2011; Thompson et al., 2017). The Harman’s single factor test revealed a total variance of 31.79%. Although this method has been criticized as insufficient (Podsakoff et al., 2003; Chang et al., 2010), it does suggest that common methods bias could have had only a negligible influence on the observed effects. Moreover, the measures were distributed between T1 and T2. We tested our hypothesized moderated mediation models with the PROCESS Macro – model 87 (Hayes, 2018). Sample bootstrapping at 5,000 was applied as recommended by Hayes (2013). In the regression models, we included gender (0 = male, 1 = female), age (at T2), academic grade [using cumulative grade point average (CGPA), which ranges from 0.0 as lowest grade and 5.0 as highest grade], and history of business in the family (0 = no, 1 = yes) as control variables. Among these controls, only history of business in the family was related to entrepreneurial intentions (*B* = 0.64, *p* < 0.05).

**Table 1:** Descriptive statistics and correlations among study variables.

S. No		*M*	*SD*	1.	2.	3.	4.	5.	6.
1.	Proactive personality – T1	5.61	1.13	**0.86**					
2.	Psychological capital – T1	5.85	0.84	0.66^***^	**0.90**				
3.	Overall family support – T2	4.78	1.01	0.21^**^	0.19^*^	**0.84**			
4.	Entrepreneurial intention – T1	5.99	1.20	0.57^***^	0.47^***^	0.18^*^	**0.90**		
5.	Implementation intention – T2	4.87	1.15	0.50^***^	0.41^***^	0.37^***^	0.51^***^	**0.92**	
6.	Entrepreneurial action – T2	3.98	1.18	0.38^***^	0.35^***^	0.33^***^	0.33^***^	0.53^***^	**0.91**

The bold values refer to Cronbach’s alpha coefficients.

* *p* < 0.05;

** *p* < 0.01;

*** *p* < 0.001.

**Figure 2:**
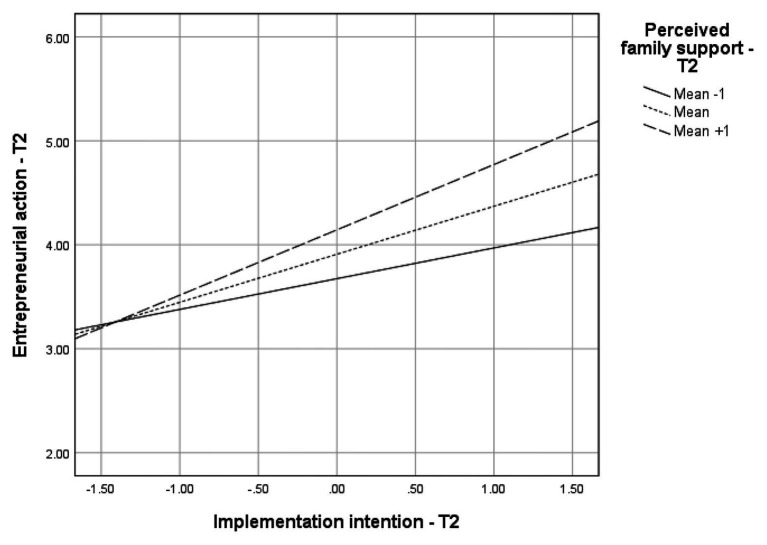
Conditional indirect effects of proactive personality on entrepreneurial action through entrepreneurial intention and implementation intention.

We predicted in Hypothesis 1 that a proactive personality would be positively associated with (a) entrepreneurial intentions, (b) entrepreneurial intention implementations, and (c) entrepreneurial actions. We also predicted a double mediation of the effects of a proactive personality on entrepreneurial actions through entrepreneurial intentions and implementation intentions (Hypothesis 3a). In support of these propositions, results of the moderated mediation analysis in Table 2 illustrate the positive effects of a proactive personality on entrepreneurial intentions (*B* = 0.52, *p* < 0.001) and implementation intentions (*B* = 0.33, *p* < 0.001). The index of the moderated mediation revealed that a proactive personality was only indirectly related to entrepreneurial actions through entrepreneurial intentions (*B* = 0.06, *Boot CI* = 0.01, 0.15), and through the double mediation by entrepreneurial intentions and implementation intentions (*B* = 0.03, *Boot CI* = <0.01, 0.08). Moreover, Hypothesis 4a suggests that perceived family support moderates the indirect effects of a proactive personality on entrepreneurial actions through entrepreneurial intentions and implementation intentions. The results further indicate positive effects of perceived family support (*B* = 0.23, *p* < 0.01) and interactive effects of implementation intentions and family support (*B* = 0.16, *p* < 0.01) on entrepreneurial actions. Conditional effects in Table 2 and the regression plots displayed in Figure 2 indicate that the effects of a proactive personality and implementation intentions on entrepreneurial actions were stronger at high levels as compared to low levels of perceived family support.

**Table 2:** Moderated mediation regression results for the effects of proactive personality on entrepreneurial action.

	Entrepreneurial intention (T1)				Implementation intention (T2)				Entrepreneurial action (T2)			
	*B*	*SE*	*CIs*		*B*	*SE*	*CIs*		*B*	*SE*	*CIs*	
			*LLCI*	*ULCI*			*LLCI*	*ULCI*			*LLCI*	*ULCI*
Gender	−0.01	0.18	−0.37	0.35	0.09	0.18	−0.26	0.44	−0.03	0.18	−0.35	0.33
Age – T2	0.04	0.03	−0.01	0.10	0.02	0.03	−0.03	0.07	0.06^*^	0.03	<0.01	0.11
Academic grade	−0.01	0.10	−20	0.19	−0.06	0.08	−0.25	0.14	−0.12	0.10	−0.31	0.08
History of business in the family	0.64^*^	0.25	0.14	1.14	−0.29	0.25	−0.79	0.21	0.31	0.26	−0.20	0.82
Proactive personality – T1	0.52^***^	0.07	0.39	0.65	0.33^***^	0.09	0.16	0.50	0.10	0.09	−0.09	0.28
Entrepreneurial intention – T1					0.32^***^	0.08	0.16	0.48	0.03	0.09	−0.15	0.21
Implementation intention (implement) – T2									0.46^***^	0.10	0.27	0.65
Perceived family support (family) – T2									0.23^**^	0.09	0.06	0.41
Implement × family									0.16^**^	0.06	0.04	0.29
Model summary	*R* ^2^ = 0.36, *F*(5, 142) = 16.22^***^				*R* ^2^ = 0.34, *F*(6, 141) = 11.91^***^				*R* ^2^ = 0.38, *F*(9, 138) = 9.27^***^			
∆*R* ^2^ due to implement × family									*∆R* ^2^ = 0.03, *F*(1, 138) = 6.82^**^			
Conditional indirect effects of proactive personality on entrepreneurial action					Mediator: entrepreneurial intention				Mediators: entrepreneurial intention and implementation intention			
					*Effect*	*Boot SE*	*Boot CIs*		*Effect*	*Boot SE*	*Boot CI*	
							*LLCI*	*ULCI*			*LLCI*	*ULCI*
Low perceived family support (mean − 1)					0.10	0.05	0.01	0.20	0.05	0.03	<−0.01	0.11
Average perceived family support (mean)					0.15	0.06	0.06	0.28	0.08	0.04	0.01	0.15
High perceived family support (mean + 1)					0.21	0.08	0.08	0.41	0.11	0.05	0.01	0.22
Index of moderated mediation					0.06	0.04	0.01	0.15	0.03	0.02	<0.01	0.08
Conditional effects of implementation intention on entrepreneurial actions									*B*	*SE*	*CIs*	
											*LLCI*	*ULCI*
Low perceived family support (mean − 1)									0.30^**^	0.09	0.11	0.48
Average perceived family support (mean)									0.46^***^	0.10	0.27	0.65
High perceived family support (mean + 1)									0.63^***^	0.13	0.37	0.89

Gender: female = 1 and male = 0. Academic grade: cumulative grade point average (CGPA) ranging from 0.0 to 5.0. History of business in the family: 0 = no and 1 = yes. CI = confidence interval.

* *p* < 0.05;

** *p* < 0.01;

*** *p* < 0.001.

**Figure 3:**
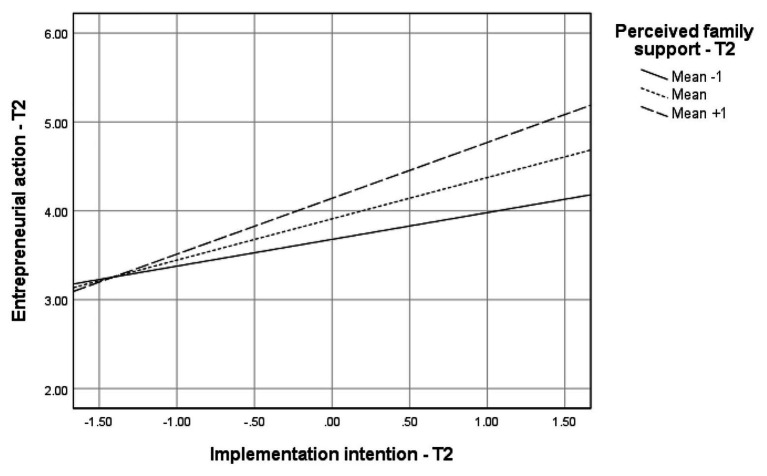
Conditional indirect effects of psychological capital on entrepreneurial action through entrepreneurial intention and implementation intention.

We further presuppose through Hypothesis 2 that psychological capital is positively associated with (a) entrepreneurial intentions, (b) entrepreneurial intention implementations, and (c) entrepreneurial actions. Similar to the findings on proactive personality, results of the moderated mediation in Table 3 show positive effects of psychological capital on entrepreneurial intentions (*B* = 0.64, *p* < 0.001) and implementation intentions (*B* = 0.29, *p* < 0.01). Psychological capital was significantly related to entrepreneurial actions indirectly (see index of moderated mediation in Table 3) through entrepreneurial intentions (*B* = 0.05, *Boot CI* = 0.01, 0.14). The double mediation by entrepreneurial intentions and implementation intentions was also significant (*B* = 0.04, *Boot CI* = <0.01, 0.11). Hence, Hypothesis 3b is supported. The significant indices of the moderated mediation analysis further show support for Hypothesis 4b that the indirect effects of psychological capital on entrepreneurial actions through entrepreneurial intentions and implementation intentions are moderated by perceived family support. The regression plots in Figure 3 illustrate that effects of implementation intentions on entrepreneurial actions are stronger at high levels of perceived family support. Similarly, the conditional indirect effects of psychological capital on entrepreneurial actions in Table 3 are stronger at high levels of perceived family support.

**Table 3:** Moderated mediation regression results for the effects of psychological capital on entrepreneurial action.

	Entrepreneurial intention				Implementation intention				Entrepreneurial action			
	*B*	*SE*	*CIs*		*B*	*SE*	*CIs*		*B*	*SE*	*CIs*	
			*LLCI*	*ULCI*			*LLCI*	*ULCI*			*LLCI*	*ULCI*
Gender	−0.15	0.19	−0.52	0.22	0.01	0.18	−0.35	0.37	−0.05	0.18	−0.41	0.30
Age – T2	0.03	0.03	−0.02	0.09	0.02	0.03	−0.04	0.07	0.05	0.03	<−0.01	0.11
Academic grade	−0.10	0.10	−0.30	0.10	−0.11	0.10	−0.31	0.09	−0.13	0.10	−0.33	0.06
History of business in the family	0.89^***^	0.26	0.38	1.40	−0.18	0.26	−0.69	0.33	0.34	0.25	−0.16	0.84
Psychological capital – T1	0.64^***^	0.10	0.44	0.84	0.29^**^	0.11	0.08	0.51	0.15	0.11	−0.07	0.37
Entrepreneurial intention – T1					0.39^***^	0.08	0.23	0.55	0.02	0.09	−0.15	0.20
Implementation intention (implement) – T2									0.47^***^	0.09	0.28	0.65
Perceived family support (family) – T2									0.23^**^	0.09	0.05	0.40
Implement × family									0.16^**^	0.06	0.04	0.29
Model summary	*R* ^2^ = 0.31 *F*(5, 142) = 12.89^***^				*R* ^2^ = 0.30, *F*(6, 141) = 10.09^***^				*R* ^2^ = 0.38, *F*(9, 138) = 9.41^***^			
∆*R* ^2^ due to implement × family									*∆R* ^2^ = 0.03, *F*(1, 138) = 6.64^**^			
Conditional indirect effects of proactive personality on entrepreneurial action					Mediator: entrepreneurial intention				Mediators: entrepreneurial intention and implementation intention			
					*Effect*	*Boot SE*	*Boot CIs*		*Effect*	*Boot SE*	*Boot CI*	
							*LLCI*	*ULCI*			*LLCI*	*ULCI*
Low perceived family support (mean − 1)					0.09	0.05	0.01	0.20	0.07	0.04	<−0.01	0.15
Average perceived family support (mean)					0.14	0.07	0.03	0.28	0.12	0.05	0.02	0.21
High perceived family support (mean + 1)					0.19	0.09	0.04	0.39	0.16	0.06	0.04	0.29
Index of moderated mediation					0.05	0.04	0.01	0.14	0.04	0.02	<0.01	0.11
Conditional effects of implementation intention on entrepreneurial actions									*B*	*SE*	*CIs*	
											*LLCI*	*ULCI*
Low perceived family support (Mean − 1)									0.30^**^	0.09	0.11	0.48
Average perceived family support (mean)									0.47^***^	0.09	0.28	0.65
High perceived family support (mean + 1)									0.63^***^	0.13	0.37	0.89

Gender: female = 1 and male = 0. Academic grade: cumulative grade point average (CGPA) ranging from 0.0 to 5.0. History of business in the family: 0 = no and 1 = yes. CI = confidence interval.

** *p* < 0.01;

*** *p* < 0.001.

## Discussion

Using the concepts of a proactive personality and psychological capital as cases of reference, the present study investigates the effects of people’s positive psychological attributes on entrepreneurial intentions and actions in the context of a developing country (e.g., Uganda). Secondly, we examined the mediating roles of entrepreneurial intentions and implementation intentions in the relationship between positive psychological attributes and actions (van Gelderen et al., 2018); and the moderating role of perceived family support in the movement from implementation intentions to taking action toward a business start-up. Our analysis demonstrates that having firm implementation intentions is essential to translating intentions into action. Moreover, even when controlling entrepreneurial intentions, implementation intentions fully mediate the effects of proactive personality and psychological capital on entrepreneurial actions. At the same time, our study illuminates that perceived family support in terms of approving the entrepreneurial activity, as well as social and financial support, plays an important role in moving from implementation intentions to engaging in actions toward creating a business start-up.

This study contributes to the understanding of entrepreneurial intention formations and its link to behavior, and entrepreneurship research in general in several ways. First, our double mediation analysis contributes to the understanding of the role of implementation intentions in the association between entrepreneurial intentions and behavior. In support of van Gelderen et al. (2018) extended model of entrepreneurial intentions, our findings show that implementation intentions mediate the effects of entrepreneurial intentions on entrepreneurial actions. Implementation intentions involve delegating the behavioral control to situational cues that consequently elicit behavior, making people become more alert to situational cues (van Gelderen et al., 2018). Consequently, people with strong intentions to implement their entrepreneurial ideas become more alert to opportunities for acting on those ideas. The full mediation of the effects of entrepreneurial intentions further suggests that having strong intentions to start a business venture without a conscious plan to implement the idea will not guarantee a start-up. Our data reveal that this seems to be the case in developing countries as well. Accordingly, implementation intentions involve both “conscious planning and automatic response activation” (van Gelderen et al., 2018, p. 927). Similarly, a strong proactive personality and high psychological capital not only translate into entrepreneurial intentions. Rather, they further enable people to consciously plan their steps of action to start a business venture and to pursue those plans whenever an opportunity arises. Hence, implementation intentions provide a good level of readiness to act. Even when opportunities arise without conscious alertness to them, it is those who have pre-planned to implement their entrepreneurial ideas who will optimize such opportunities. While implementation intentions develop out of strong entrepreneurial intentions (van Gelderen et al., 2018), our results demonstrate the strong direct effects of a proactive personality and psychological capital.

Second, we then focus on perceived family support as a way of examining the preconditions for movement from entrepreneurial intentions and implementation intentions to action. It has been posited that implementation intentions provide the motivation and commitment for action (Ajzen et al., 2009; Tornikoski and Maalaoui, 2019) and draw one to become alert to cues and opportunities in the environment (Gollwitzer, 1999; van Gelderen et al., 2018). At the same time, they provide an opportunity to assess the feasibility of the entrepreneurial idea or one’s compatibility to the entrepreneurial career path (van Gelderen et al., 2018). There are, however, several other factors that condition engagement in entrepreneurial actions toward a start-up. These are summarized in the construct of perceived behavioral control (Ajzen, 1991, 2002) as applied to entrepreneurship. Key questions in an entrepreneurial sense may arise in relation to the ability to meet start-up capital requirements and support from significant others. Beyond the known influence of family on career preferences and choices, family members and friends are a common source of capital for small business start-ups in most African communities, particularly in the informal sector. An added importance of the family for entry into entrepreneurship in the African context is the parental attitudes toward going into business after graduating from university. Many parents struggle in supporting their children through university education with their meager resources. This is done for the purposes of gaining salaried or white-collar employment in a prestigious organization. This defines career success within their local context. However, career success no longer strictly lies in a traditional organizational employment or one’s learned trade (Arnold, 2001; Baruch, 2004). Our results suggest that soliciting parental support for the entrepreneurial aspirations of their children is useful to the fruition of entrepreneurial intentions. An implementation intention is likely to elicit commitment to performing a behavior (Ajzen et al., 2009), but the presence of approval in the form of moral, social, and financial support from a Ugandan family and significant others will likely heighten the commitment to engage in start-up activities.

Third, we investigated positive psychological attributes as precursors of entrepreneurship. Our findings demonstrate the importance of people’s positive attributes when it comes to the formation of entrepreneurial intentions and their translation into actions toward an entrepreneurial start-up. While positive psychology, specifically the construct of psychological capital, has been applied to explaining entrepreneurs’ well-being and entrepreneurial outcomes (Jensen and Luthans, 2006; Baron et al., 2016; Baluku et al., 2018a; Bockorny and Youssef-Morgan, 2019), it has not received enough attention within entrepreneurial intentions research (Jin, 2017; Baluku et al., 2019c). Our study demonstrates that positive psychological attributes have strong effects on entrepreneurial and implementation intentions even in developing countries where the environment is often not that supportive. Moreover, both a proactive personality and psychological capital have strong direct effects on implementation intentions even when entrepreneurial intentions were added to the model as a mediator. Our results further indicate that the two positive individual attributes have similar effects on entrepreneurial intentions, implementation intentions, and entrepreneurial actions. The effects of both attributes on entrepreneurial actions are fully mediated by entrepreneurial intentions and implementation intentions.

People with a strong, proactive personality have the predisposition to exhibit proactive behaviors including, but not limited to, creativity, networking, and perseverance (Crant, 1996; Fuller and Marler, 2009). Similarly, psychological capital comprises positive psychological resources including confidence, hope, optimism, and resilience (Luthans et al., 2004, 2015). Such attributes are essential for taking entrepreneurial actions as they involve the ability to construct goals and plans, developing alternative ways of achieving goals, and persevering in pursuing those goals, respectively. These attributes relate to what is described as constituting implementation intentions (Gollwitzer, 1993, 1999). Hence, it should not be surprising that implementation intentions fully mediate the effects of the two positive psychological attributes and entrepreneurial intentions on entrepreneurial actions.

## Limitations

At least two limitations should be mentioned. First, the study was conducted with students enrolled in only one program as participants. Hence, the program, being related to management studies, already introduced students to business concepts before the entrepreneurship course. This may partially account for the high entrepreneurial intentions at T1, which might have produced a ceiling effect. This effect could have also diminished the chance of finding a strong association between intentions at T1 and action at T2. Nonetheless, it is to be noted that previous studies have shown that the overall level of entrepreneurial intentions in Uganda compared to other countries is comparatively high (GEM, 2014) so that high entrepreneurial intentions can also be perceived as characteristic for the country. Future studies on entrepreneurial intentions and actions of students should consider using multiple groups in regard to program of study, length of study (those at the beginning vs. those at the end of the study program), and the context (small vs. big town and country contexts).

Second, although the use of a longitudinal approach is certainly a strength of this study, the period between the two measures was only 4 months. The T1 assessment was taken at the start of the course, while the T2 assessment was taken shortly after the end of the course. This means that at the end of the course, students may still have been excited about becoming entrepreneurs without practically observing realities in the environment that may limit or facilitate actions. This could have resulted in the reporting of high implementation intentions. Moreover, participants responded to all the measures. Although the Harman’s single factor method reveals that this was not a major concern, we cannot rule it out with certainty. In addition, entrepreneurial implementation intention behavior may form over a long period of time. The short follow-up period limits the analysis of the success of the actions geared toward start-up. It is also possible that some participants already engaged in some actions toward entrepreneurial start-up, which we did not include in our control measures. Hence, future studies should consider measuring implementation intention and action after a long period of time while considering drawbacks; and control for entrepreneurial actions taken before enrolling for entrepreneurship education programs. Moreover, it might be useful to measure implementation intentions and actual behavior at different times and not concurrently.

## Practical Implications

In practical terms, our results highlight the importance of supporting prospective entrepreneurs beyond developing a strong intent to go into business. There should be deliberate interventions to support prospective entrepreneurs to develop realistic plans and steps to undertake the implementation of their entrepreneurial ideas. This may help in overcoming what Van Gelderen et al. (2015) refer to as action aversion. Moreover, it is important to continuously support prospective entrepreneurs to strengthen their proactive behaviors and psychological resources.

In addition, the study highlights the role and importance of entrepreneurship education at university or college. Our results indicate that entrepreneurial education is effective in supporting students translate entrepreneurial intentions into actions geared toward a start-up. However, interest should be taken to understand at what stage of university education is entrepreneurship education most effective. The present study focuses on evaluating entrepreneurship education that was offered to students in their final semester. This may be effective given that students at this stage are already engaged in the school-to-work transition (Baluku et al., 2020), and hence able to consider entrepreneurship actions as employment alternatives. On the other hand, entrepreneurship education offered at the start of the university education may offer educators to support incubation of students’ entrepreneurial ideas for a longer period. Moreover, a common challenge to entrepreneurship education in developing countries (at least in the case of Uganda) is the tendency to examine entrepreneurship courses theoretically. This limits the practical focus of entrepreneurship education. At the policy level, our findings suggest that there is need to design entrepreneurship courses to be more practical and less academic oriented. That is, entrepreneurship course should be oriented toward innovation and nurturing ideas than focusing on preparing students for entrepreneurship exams.

The study results further suggest that training and educational programs aimed at stimulating entrepreneurial start-ups should incorporate efforts to boost proactive behaviors and psychological resources. Similarly, Campos et al. (2017) demonstrated that focusing on positive psychological attributes yields superior results than traditional or generic entrepreneurship training. Specifically, Luthans and Youssef-Morgan (2017) provide several interventions that can improve psychological capital, which includes improving self-efficacy, hope, optimism, and resilience, for example through supporting students develop smart goals, challenging them to engage in positive actions on a daily basis, coaching, and specifically tailored innovative games. Whereas these are interventions for improving psychological capital, they can also support in the development of proactivity. Proactivity can also for example be improved through challenging students to develop smart goals, identify barriers to obtaining the set goals and how to overcome the barriers, coaching and mentoring especially through practical exercises, and innovative games.

Furthermore, it seems critical to foster an appreciation for entrepreneurship as a feasible career path among not only young people but also parents and other family members. This, especially, given the small odds of achieving a formal employment in countries with extraordinarily high youth unemployment rates, such as Uganda. Entrepreneurship awareness and promotion interventions in developing countries tend to primarily focus on youth. Expanding the scope of target groups and awareness among parents, teachers, and other people who play significant roles in career choices and decisions of children have the potential to increase the number of young people starting their own enterprises.

Family support is important to entrepreneurial intention and actions of students in countries like Uganda where young people have limited access to start-up materials. As indicated in the literature, the family is often the source of start-up resources in addition to psychological and emotional support. In terms of policy therefore, governments and universities should consider providing incentives for student innovations that can support the incubation of novel entrepreneurial ideas. It is essential for universities in developing countries to invest resources in incubation centers for students’ entrepreneurial ideas. Both universities and governments should also consider moving toward creating entrepreneurial universities (Lazzeroni and Piccaluga, 2003; Wong et al., 2007). This can be done through interventions such as entrepreneurship and innovation competitions, offering innovation grants, collaboration with industry in the development of entrepreneurship skills as well as incorporating entrepreneurship and entrepreneurial mind-set related content in most of the study programs. Our study has indicated that entrepreneurship education for non-business students can stimulate entrepreneurship action.

## Conclusion

This paper contributes to the current debate on how the gap between entrepreneurial intentions and entrepreneurial action can be bridged. Based on the implementation intention model (Gollwitzer, 1993, 1999), our Ugandan findings demonstrate that the relationship between antecedent factors and entrepreneurial action occurs through a double mediation of goal intentions (entrepreneurial intentions) and implementation intentions. Our study reveals that perceived family support increases the likelihood of entrepreneurial intentions and implementation intentions, translating into actual start-up actions. These findings are particularly relevant for developing countries, such as Uganda, where data show a striking difference between rather high levels of entrepreneurial intentions and the relatively low early-stage entrepreneurial activities (GEM, 2014; Singer et al., 2015).

## Data Availability Statement

The raw data supporting the conclusions of this article will be made available by the authors upon request, without undue reservation.

## Ethics Statement

Ethical review and approval was not required for the study on human participants in accordance with the local legislation and institutional requirements. The patients/participants provided their written informed consent to participate in this study.

## Author Contributions

MB was responsible for drafting the manuscript. JK played an important role in implementing the intervention. CK, KO, and NB played important roles in the conceptualization process and in the writing of the manuscript. All authors contributed to the article and approved the submitted version.

### Conflict of Interest

The authors declare that the research was conducted in the absence of any commercial or financial relationships that could be construed as a potential conflict of interest.
